# Environmental risk assessment of GE plants under low-exposure conditions

**DOI:** 10.1007/s11248-013-9762-z

**Published:** 2013-11-01

**Authors:** Andrew Roberts, Yann Devos, Alan Raybould, Patrick Bigelow, Alan Gray

**Affiliations:** 1 Center for Environmental Risk Assessment, ILSI Research Foundation, Washington, DC USA; 2 GMO Unit, European Food Safety Authority (EFSA), Via Carlo Magno 1, 43126 Parma, Italy; 3 Syngenta, Jealott’s Hill International Research Centre, Bracknell, UK; 4 Plant Breeding, Genetics and Biotechnology, Michigan State University, East Lansing, MI USA; 5 Center for Ecology and Hydrology Wallingford, Wallingford, UK

**Keywords:** ERA, Agricultural biotechnology, Low exposure, Risk assessment

## Abstract

The requirement for environmental risk assessment (ERA) of genetically engineered (GE) plants prior to large scale or commercial introduction into the environment is well established in national laws and regulations, as well as in international agreements. Since the first introductions of GE plants in commercial agriculture in the 1990s, a nearly universal paradigm has emerged for conducting these assessments based on a few guiding principles. These include the concept of case-by-case assessment, the use of comparative assessments, and a focus of the ERA on characteristics of the plant, the introduced trait, and the receiving environment as well as the intended use. In practice, however, ERAs for GE plants have frequently focused on achieving highly detailed characterizations of potential hazards at the expense of consideration of the relevant levels of exposure. This emphasis on exhaustive hazard characterization can lead to great difficulties when applied to ERA for GE plants under low-exposure conditions. This paper presents some relevant considerations for conducting an ERA for a GE plant in a low-exposure scenario in the context of the generalized ERA paradigm, building on discussions and case studies presented during a session at ISBGMO 12.

## Introduction

Risk has historically been expressed as a function of two components: hazard and exposure. Hazard is the inherent property of a thing, or of an action that might lead to harm (e.g. toxicity), while exposure is the measure of how much interaction occurs between the hazardous thing or action and a specific entity (usually one that is protected or valued). This description of risk works well for simple cases with easily understood hazards and easily measurable exposures, but it is sometimes difficult to conceptualize for more complex cases. For environmental risk assessments (ERA) of genetically engineered (GE) plants, it is useful instead to consider risk as a function of the likelihood of an environmental harm and the consequences, or magnitude of that harm (Hill 2005; Raybould 2006; OGTR 2009; Wolt et al. 2010). ERA is informed by the generation and testing of plausible risk hypotheses, which are derived from a risk scenario detailing the necessary steps or interactions that are required for the GE plant to cause harm in the environment. The likelihood of a harm being realized is dependent on the likelihood of each step in the risk scenario. When evaluating the likelihood and seriousness of harm to the environment following the cultivation of a GE plant, the ERA assumes 100 % exposure over an extended period of time. Exposure and potential impact are expected to be the highest under cultivation conditions. However, under low-exposure scenarios, the context usually differs, as only few GE plants are present in the environment. Therefore, low-exposure scenarios can be expected to reduce the likelihood of one or more steps in a risk scenario, compared with a cultivation scenario.

For a variety of reasons, ERA for GE plants has often focused on exhaustive characterization of the differences between the GE plant and a non-transformed counterpart, without considering whether these differences affect the likelihood or consequences of an environmental harm. There are a number of possible explanations for this, including the presumed high level of exposure for GE plants introduced as agricultural crops which will be cultivated on large acreages over multiple years, as well as difficulty in defining environmental harms (Sanvido et al. 2012). Certainly in the case of ERA conducted in support of approvals for large-scale cultivation a full understanding of potential hazard is warranted, and identifying relevant differences between the GE plant and a non-transformed counterpart may help identify potential hazards. However, it is increasingly common for ERA to be conducted for GE plants that are expected to be introduced into the environment at low-exposures. Although it is generally acknowledged that low-exposures will have a significant impact on the likelihood of harm, there is little practical guidance for conducting ERA under low-exposure scenarios.

This paper builds upon the presentations and discussions at a session on environmental risk assessment under low-exposure scenarios conducted during the 12th International Symposium on the Biosafety of Genetically Modified Organisms (ISBGMO 12) held in September 2012 in St. Louis, Missouri. Posing the question, “what, if anything, should be done differently when conducting ERA for releases of GE plants under low-exposure scenarios?” we consider the generalized ERA for GE plants, the nature of potential low-exposure introductions, and present a stepwise approach to conducting ERA under situations of low-exposure.

## The generalized ERA paradigm for GE plants

The first GE plants were approved for cultivation in the mid-1990s, and these approvals were informed by ERAs. At present, most countries have domestic regulations requiring ERA prior to the release of a GE plant, and although these assessments differ in scope and form, they typically consider similar potential hazards. These include the possibility that the GE plant, or any wild relative receiving the transgene via gene flow, may become a weed in an agricultural environment or invasive of natural habitats leading to harmful reductions in species or population abundance either through competition or harmful impacts of the introduced gene (Chandler and Dunwell 2008; Lu 2008).

Significant experience has been accrued in both the assessment and cultivation of GE plants and, although there is no formal international standard for ERA, a clear consensus paradigm can be discerned. The paradigm consists of a risk assessment framework as well as a set of common principles that are applied to that framework. The risk assessment framework has been variously described but can be divided into four essential steps. The first step establishes the context for the assessment, incorporating the legislative, policy and regulatory goals that are relevant for the decision being informed by the assessment. This defines the scope of the assessment and establishes what should be considered and how it should be considered. The second step involves the identification of risk scenarios and plausible pathways to harm based on the activity being proposed and the context established in step 1. These two can be grouped together and referred to as “problem formulation.” This is followed by characterization of the likelihood of harm being realized, as well as characterization of the potential magnitude and severity of adverse outcomes (step 3). These are then combined to provide an overall estimate of risk (step 4). Depending on the jurisdiction, consideration of risk management options for any identified risks may or may not be considered to be formally part of the risk assessment, but it is always part of the overall risk analysis related to decision making.

In addition to a common risk assessment framework, there are common principles that are also part of the generalized ERA paradigm for GE plants. First, assessments take into account the biology of the plant, the characteristics of the introduced trait, the characteristics of the receiving environment and the interactions between all three. The relevant biology of the plant normally includes the growth and reproductive habit and is essential for understanding the potential for survival and persistence of the GE plant. The characteristics of the trait include the introduced gene or genes as well as the protein or other gene products they produce along with the resultant phenotype in the plant. The receiving environment refers to the environment where the GE plant will be introduced and normally includes consideration of management practices and intended use of the plant, as well as the potential for the plant to survive and persist within, or to move outside, the environment where it is intended to be used. Second, assessments are comparative, typically comparing the GE plant to its untransformed counterpart. The reason for this is that assessments are not intended to identify all the risks associated with agriculture, but rather to identify any additional risk that will be conferred by the introduction of the GE plant under consideration. Finally, assessments are conducted case by case. This means assessments are conducted on specific plants in specific situations, but it doesn’t mean that assessments must start from scratch. The use of relevant existing knowledge, including from previous assessments of other GE crops, informs the problem formulation and the identification of plausible risk hypotheses.

## Low-exposure scenarios

Precisely what constitutes low-exposure is a subjective question. However, several situations commonly occur that typically are treated as low-exposure scenarios. It is worth considering these scenarios in detail, because they will offer different challenges for risk assessors.

### Field trials

One area where risk assessors and regulators have significant experience in considering ERA for GE plants under low-exposure scenarios is in the conduct of small-scale, confined field trials for experimental purposes. A field trial represents a deliberate introduction of a GE plant into the environment under low-exposure conditions (OECD 1993). Although all of the plants in a given field will be GE, exposure is low because of the size of the trial in the context of the agricultural environment and because of the management measures employed to confine the trial plant geographically and temporally within the trial site. It is instructive to consider how field trials are managed and how regulatory agencies make decisions to allow them in the absence of exhaustive hazard characterization, which is typically not available.

Although conditions and risk management measures applied to field trials vary between jurisdictions they typically share the common goals of ensuring that the GE plant does not survive and persist outside of the spatial and temporal limits of the trial. Usually, specific conditions are imposed to limit the spread and persistence of the GE plant or the introduced genes, including isolation distances and buffer strips to minimize outcrossing with the conventional counterpart or hybridization with sexually cross-compatible wild relatives; confinement measures to avoid the occurrence of volunteer plants in subsequent years originating from spilled seeds and/or vegetative plant parts during the trial and mixing of plant material in machinery during sowing, harvest and/or post-harvest operations; post-release follow-up to control potential volunteer plants occurring in subsequent years; and crop destruction. In most cases, plant material from these trials cannot enter the food and feed chain and has to be destroyed at the end of the trial.

### Unintended environmental introductions of grains imported for food and feed

A country may approve a GE plant for use as food or feed (including import of the GE plant and derived products from an exporting country where the GE plant is grown, and processing in the importing country), but not for introduction into the environment for cultivation. Depending on the plant species, spillage of grains during transport may lead to transient or established populations of feral plants growing typically along roadsides or other disturbed habitats surrounding shipping centers such as ports or railroad stations (Bagavathiannan and Van Acker 2008). Under this scenario the percentage of plants in the population of escaped plants which are GE could be very low or could be 100 %. In either case, the exposure would be considered low because the area where the plant occurs, and the number of plants would typically be small (Devos et al. 2012).

### Low level presence in seed and grains

Regulatory approvals for GE plants are conducted at the national or regional level, but the production and distribution of seed for planting is an increasingly international activity. It is therefore possible for a GE event that has not been approved in the country of destination to be co-mingled with seed that is authorized for use in the country of destination (either because it is intended to be non-GE or contains another GE event that is approved in the country of destination). This is sometimes referred to as low level presence or LLP (OECD 2013). LLP can occur because of human error, cross pollination during seed production or breeding, admixture during shipping or a combination of these factors. Similar exposure scenarios can be envisioned in domestic seed that has low levels of unapproved GE events acquired from field trials or breeding programs conducted within a country. Further, small amounts of unauthorized GE events may occur in shipments of grain for use in food or feed (including import and processing). For ERA purposes, these scenarios represent an exposure to the environment of an unauthorized GE event for which information to support robust hazard characterization may or may not be available. Although there are no formal threshold levels defining what constitutes LLP, experience suggests that occurrences will be typified by low percentages of unauthorized seed introduced into agricultural fields sown with large numbers of related plants.

## ERA for GE plants under low-exposure scenarios

As for all ERA of GE plants, assessments conducted under circumstances of low-exposure must be conducted case by case. The specific details of the plant biology, introduced trait and receiving environment will continue to have a profound effect on the outcome of the assessment. However, there are some aspects of ERA under low-exposure scenarios that may be generalizable and it is useful to think about these in the context of the existing risk assessment paradigm and in the context of the scenarios previously identified.

### Problem formulation for ERA under low-exposure scenarios

The first part of problem formulation incorporates relevant laws and regulations, as well as the societal values these represent, in order to identify what aspects of the environment are valued and should be protected. The environmental protections goals thus identified are then considered in the context of the specific case to identify which ones are likely to be relevant. Environmental protection goals vary between countries, but those that are relevant to large-scale introductions of GE plants will also be relevant to introductions at low-exposure. In this respect, at least at the broadest level, the ERA is not impacted by the level of exposure. However, as the problem formulation continues to consider plausible risk hypotheses and to identify the components of an analysis plan, the consideration of exposure may impact those risk hypotheses which are considered plausible, and may impact the selection of assessment endpoints.

### Learning from field trial management

The most common scenario for the low-exposure introduction into the environment of a GE plant is a confined field trial. These are routinely conducted, even in many jurisdictions where approvals for cultivation of GE plants are rare or nonexistent. Because the potential hazard is unknown, or poorly characterized, field trials are subject to intense management. Normally, field trials are permitted based on an understanding of the ability of the unmodified plant to survive and reproduce in the receiving environment and in the context of defined management strategies. This is coupled with the understanding that sufficiently low-exposure makes adverse outcomes unlikely for all but the most extreme hazards (Hill 2005; OGTR 2009). Decisions regarding field trials are typically not based on exhaustive characterizations of the GE plant, although varying amounts of information and experience related to the introduced trait may be available. Instead, they focus on ensuring that, considering the biology of the plant and the environment where the trial is occurring, management practices will be sufficient to keep the trial confined, temporally and spatially. In other words, in the face of an uncertain hazard, the assessment focuses on whether or not the proposed trial conditions are adequate to maintain the low-exposure which is intended.

Thus, there is a precedent for ERA under low-exposure conditions where information on the hazard may be unavailable or incomplete. In such circumstances, the risk is considered negligible if the plant occurs in a small area and is unlikely to spread and persist in the environment over time, limiting exposure both spatially and temporally. For field trials, these conditions are created through management measures, but under other low-exposure scenarios the ability of the GE plant to spread and persist in the environment may be limited by the nature of the introduction, factors in the receiving environment, and the biology of the plant. This suggests that ERA can proceed in a stepwise manner where first the assessor considers whether, under the conditions of the specific low-exposure scenario being addressed, the GE plant is likely to spread or persist in the environment and whether this would lead to an increased exposure. If not, then the assessor may be able to conclude that the likelihood of environmental harm being realized would be low even in cases where exhaustive characterization of the plant is unavailable, and in the absence of some information typically required for ERA, such as evaluation of impacts to non-target organisms (NTOs).

### ERA for unintended introductions of grains intended for food and feed

The unintended introduction of GE plants to the environment in the context of spills or accidental releases of grain that is intended for food or feed is different form the release of GE plants intended for cultivation in two primary ways. First, the environmental exposure is much lower because the area of introduction is small, and second, the receiving environment is generally a disturbed habitat near a transportation center or route rather than an agricultural environment. In addition, there is potentially tremendous variability in both the amount of seed introduced as well as the ability of the seed to germinate and produce viable plants (Bagavathiannan and Van Acker 2008). In these scenarios, the receiving environment will greatly impact the assessment and should be considered during problem formulation for the ERA, particularly in the identification of relevant protection goals. While there will certainly be some overlap, the types of disturbed habitats where spilled seeds persist are generally not regionally sensitive or protected areas and, because they are typically disturbed by human activities regularly, are unlikely to provide reliable habitat for protected species or significant ecological services. The overall area of these types of habitats is generally small, and the exposure to the overall environment from a GE plant introduced can therefore be considered low. The assessment should therefore consider ERA for a GE plant released in this way in the context of the environmental protection goals and management strategies that are generally employed in the receiving environment (Devos et al. 2012).

As with considerations for field trials, an important component of the assessment will be consideration of whether or not the GE plant will persist in the environment as well as its potential to spread to other environments. Although detailed characterizations of the GE plant in the specific receiving environment will not be available, there is likely to be information from the country of origin which can be used to determine if the GE plant has significantly altered characteristics when compared to its conventional counterpart. If differences in the relevant biological characteristics for determining persistence and spread in the environment have not been observed, then available information and experience with the unmodified plant can be directly applicable to the ERA. In addition, a wealth of information may be available from previous experience with the introduced trait (Center for Environmental Risk Assessment 2011, 2012a, b, c).

### ERA for low level presence in seed and grain

For ERA of LLP under conditions of cultivation, the receiving environment will be agricultural. Depending on the specific case, the cumulative number of plants involved may be quite large, but environmental exposure will be diffuse, by definition. Once again, under these conditions a GE plant would have to represent a very serious hazard to pose a significant risk to the environment. Furthermore, crops are planted for the purpose of harvesting so the exposure would be both spatially and temporally isolated. The important question for risk assessment then becomes whether or not the exposure is likely to remain low, or if the GE plant will persist and multiply in the agricultural environment.

In all of the scenarios considered above, the fundamental risk equation dictates that sufficiently low-exposures lead to minimal or negligible risks independent of the specific details of any particular hazard, and this is supported in regulatory assessments and guidance for GE plants (EFSA 2010; Devos et al. 2011). However, any ERA conducted for a GE plant under low-exposure conditions would need to determine if the exposure is likely to remain low.

### A stepwise approach for ERA under low-exposure scenarios

Based on the experience with ERA for GE plants described above, it is proposed that a stepwise approach is appropriate for future assessments. When a GE plant is determined to be present in the environment at low levels, and assessment is required, the first step is to determine if the plant is likely to survive and persist in the environment. If not, then there is little need for further assessment since the low levels of exposure are unlikely to lead to harm. If the plant will survive and persist in the environment, the second step would be to evaluate whether that persistence will lead to future increase of exposure to the GE plant in the receiving environment. If the exposure is likely to remain low, then it will remain unlikely that environmental harm will be realized. However, if an increase in exposure is plausible, then additional information may need to be considered to determine the risks including additional characterization of the potential interactions of the plant with flora and fauna.

The following sections provide some guidance on what information would be valuable for determining if a GE plant is likely to survive and persist in the environment, and if persistence is likely to lead to increased exposure through the spread of the GE plant into the surrounding environment. In addition, potentially useful sources of information are discussed, as some of the information needed to adequately assess the environmental risk of low-exposure scenarios with a GE plant may already be available and come from existing knowledge and experience with the plant species, trait and receiving environment (OECD 2013).

### Information for determining likelihood of survival and persistence

#### Biology and reproduction of the plant

Knowledge of the reproductive biology of the parental species, including all the modes of reproduction, dissemination and survivability, is likely to be informative when considering the probability that the plant will survive or persist in a particular environment. Characteristics to consider may include the relative vigor of the plant, as well as the ability of seed or other propagules to disperse, survive and propagate. For exposures which occur in the context of agriculture or other heavily managed ecosystems, the ability of the plant to present as a volunteer and of seed to remain viable over time (i.e. seed dormancy) will be important considerations for determining the duration of exposure. Data on the characteristics of the parental species is widely available for most major crops and summary biology documents produced by governments or international organizations such as the OECD provide a valuable resource (CFIA; OECD; OGTR).

#### Potential for weediness or invasiveness

The characteristics that make plants weedy or invasive are well aligned with those that allow them to survive and thrive in a particular environment (Pheloung et al. 1999; Richardson et al. 2000). Considering the potential of the parent plant to be weedy or invasive will provide insight into whether or not the GE plant under assessment is likely to survive and persist in the environment. Although characteristics associated with weediness or invasiveness have been bred out of many crops during domestication, the degree of domestication varies by crop and it is important to account for this in the ERA (Warwick and Stewart 2005; Keese et al. 2013). Some domesticated species may still contain weedy or invasive characteristics such as seed dormancy, discontinuous germination, rapid seedling growth, phenotypic plasticity, asynchronous flowering, shattering and other seed dispersal mechanisms, or strong competitive ability (Warwick et al. 2009). The history of cultivation of the parental species can be examined for evidence of whether these plants have become a weed or invasive, either in the receiving environment under consideration or elsewhere. Data collected in field trials and associated with regulatory approvals can be useful to assess whether the GE plant under assessment has altered characteristics related to survival and persistence. This frequently includes field observations for volunteer populations as well as measurements of seed dormancy along with a comparative assessment of growth habits to the unmodified parent. Experience from wide-scale cultivation in other countries may also be a useful source of information for determining whether the GE plant has altered survival characteristics.

#### Factors limiting weediness or invasiveness

Many abiotic and biotic factors limit the ability of plants to form self-sustaining populations under either cultivated or uncultivated conditions. It is therefore relevant to describe factors that may restrict or limit the niche of the plant to certain habitats, or that may control its population size, according to the current state of knowledge. This can be useful for assessing whether the GE plant is likely to behave differently with respect to any of these factors, and for determining whether or not the plant is likely to survive or persist in the environment.

#### Potential for gene flow, hybridization, and introgression

Since genetic material can move spatially and temporally through the transfer of pollen, seeds, or vegetative propagules, the assessment should consider relevant avenues and vectors for gene flow, together with factors that affect the probability of these processes. These include knowledge on the presence of wild relatives in the receiving environment, the potential to hybridize with sympatric compatible relatives, and the ability of hybrids to persist or cross back into either parental species. If the transgene from the GE plant is able to persist through introgression into persistent populations of sexually compatible relatives, then the risk assessment will need to consider the potential for an increase in exposure due to the presence of the gene in the persistent population. The assessment should also fully consider factors which limit the probability of successful hybridization such as proximity, flowering synchrony or genetic compatibility issues. Gene flow, hybridization and introgression are not unique to GE plants (Ellstrand et al. 1999, 2013), and any history or experience with the parental plant will be informative for determining whether there is the potential for gene flow that might affect the likelihood of environmental harm. It is important to note that exhaustive quantification of gene flow is not required (Raybould 2006, 2010).

#### Information on the characteristics of the GE plant in comparison to the untransformed counterpart

The ERA would also need to consider whether the GE plant is similar to the untransformed counterpart with respect to the characteristics identified as being important for survival and persistence in the environment. If data indicates changes in these characteristics, those changes would need to be assessed for their potential to alter the likelihood of survival and persistence of the GE plant. Depending on the specific context of the risk assessment, more or less comparative data may be available. However, during development of a GE plant the types of data indicated below are typically collected.

- Phenotype under agronomic conditions
  The general phenotypic and agronomic characteristics of the GE plant are normally assessed in multi-location field trials representative of the different environments where the GE plant may be grown in order to establish intended or potential unintended differences between the GE plant and its conventional counterpart (Horak et al. 2007; Raybould et al. 2010, 2012). Characteristics under consideration typically include plant establishment and vigour, growth, plant height and dry matter production, seed and yield characteristics, vernalisation requirement, and morphology. In addition, visual observation related to the plants response to insects, disease or other stresses are generally also recorded.
- Reproductive biology
  Data on reproductive biology are normally collected through observations of field trials and may include observations of time to flowering and maturity, attractiveness to pollinators, and pollen shed, viability and compatibility. In addition, laboratory experiments are sometimes performed to assess pollen viability.
- Seed persistence and germination
  Growth chamber experiments or information collected during field trials normally enable the assessment of seed germination and other dormancy characteristics of the GE plant under various conditions. Measurements or observations such as volunteer number in subsequent crops/plantations indicate the potential for seeds and vegetative propagules from a GE plant to give rise to volunteer populations. Post-harvest field inspection data in which volunteer numbers are reported can serve as an information source and provide indications on the overwintering potential of the GE plant seeds. Seed burial experiments can also give indications of changes in dormancy and seed persistence (Hails et al. 1997).

The types of information described above are likely to be available to risk assessors faced with a low-exposure scenario. The majority of low-exposure scenarios (excluding field trials for experimental purposes) have involved ‘common’ crop plant species and trait combinations that have been widely adopted and are under large-scale cultivation where approved. Therefore, there is substantial knowledge and experience with these plant species and the newly expressed traits as they are grown regularly within countries in which low-exposure situations can occur (OECD 2013).

### Information for determining the potential for increased exposure

If the assessment concludes that there is a reasonable probability that the GE plant being assessed will survive and persist in the environment, either because the GE plant itself will persist or the transgene is likely to introgress into compatible populations, the next step is to determine if this persistence is likely to remain at low levels, or if there is potential for the exposure to increase.

#### Fitness effects of the transgene in relevant populations

Fitness is variously defined, but it is generally considered to be a measure of how likely an individual (or a genotype) is to contribute offspring to subsequent generations. For the purposes of risk assessment under conditions of low-exposure it may be important to estimate the relative fitness contribution of the transgene, since this will determine whether or not the frequency of the allele increases, decreases, or remains the same over time. If the transgene is shown to have a fitness benefit, then it can be expected that the frequency of the transgene will increase in the population. The ERA will then need to consider the consequences of this increase in the context of both the population and the low-exposure scenario. For example, if the transgene frequency were to rise to 100 % in a small population of plants growing in a ruderal environment (e.g. a roadside ditch near a commodity transportation facility), then this increase would have little consequence in terms of increasing the overall exposure to the environment. However, if the frequency of a transgene were to increase in a substantial naturalized population then this might have a significant effect on the overall exposure to the environment, and would necessitate further assessment of the consequences of that increase in exposure. It is important to understand that an increase in exposure does not necessarily mean that harm will occur. Any subsequent assessment would need to consider the consequence of the gene flow, including the potential for impacts on other species.

#### The potential for the trait to lead to ecological release

The ecological release concept originates in the study of invasive species, and generally asserts that the population size of any species is constrained by one or more factors (Schmitt and Linder 1994; Wilkinson and Tepfer 2009). If enough of these factors are removed—either by introducing the species into a new environment or by significantly altering the native environment, then the population may expand. ERA for GE plants under low-exposure scenarios will have to consider the likelihood that the introduced trait could contribute to the ecological release of a persistent population. Although similar to the concept of fitness, under an ecological-release scenario the gene would not only increase in frequency in the population, it would also expand the size and geographic distribution of the population. In order to assess the likelihood of ecological release, information on persistent populations and their ecological interactions would be useful, especially any history of invasiveness in other receiving environments. In that context, consideration should then be given to whether or not the transgene in the GE plant is likely to alter the plant in a way which affects a control factor.

## Conclusions and recommendations

Environmental risk assessments under conditions of low-exposure can be conducted using the same general paradigm for risk assessment as other ERA for GE plants. In particular, the protection goals established through laws and regulations and identified as relevant for GE plants will not change. However, the low-exposure may not necessitate the kind of extensive characterization of potential hazard that normally accompanies risk assessment for large-scale environmental introductions, such as releases for commercial cultivation. If a plant or transgene is introduced at low levels, and is not expected to persist and multiply in the environment, thereby increasing the level of exposure, then risk will likely remain low. For this reason, the following stepwise approach for ERA of GE plants under low-exposure conditions is recommended.

### *Step 1:*

*Initial characterization of the GE plant, trait and environment in the low*-*exposure scenario*

It is important to consider the context of the low-exposure. The introduction of a small percentage of GE plants in the context of cultivation will be significantly different than the introduction of a high percentage, but small numbers, of GE plants into disturbed habitats such as roadsides as a result of transportation. Further, the nature of the exposure will determine which protection goals might plausibly be connected to the introduction through risk hypotheses. For example, spatially isolated populations in urban areas (such as around ports) are unlikely to pose a risk to valued entities in the agricultural or other rural landscapes.

### *Step 2:*

*Asses the likelihood that the GE plant establishes, reproduces and disperses*

These characteristics are determined by inherent properties of the plant and can normally be informed through a review of literature and comparison with the untransformed plant. Only in cases where there is a lack of familiarity with the unmodified plant in the receiving environment, or evidence suggests the GE plant is substantially different from the unmodified plant with respect to survival and persistence, would the collection of experimental data be necessary to inform the assessment.

### *Step 3:*

*If the GE plant (or transgene through gene flow, hybridization or introgression) is likely to persist, consider the likelihood that exposure will increase significantly*

If the GE plant (or transgene) is in a persistent population, the ERA will need to consider whether the exposure is likely to increase—either through an increase in the gene frequency within the population or an increase in the population itself. If the exposure is likely to remain low, then further characterization of the risk is likely not necessary. However, if the exposure is expected to increase, then the assessment would need to consider the nature of that increase, and would likely need to expand to consider additional characterization of the GE plant and its interactions in the environment.

### Using the stepwise approach for ERA under low-exposure scenarios

**Fig. 1:**
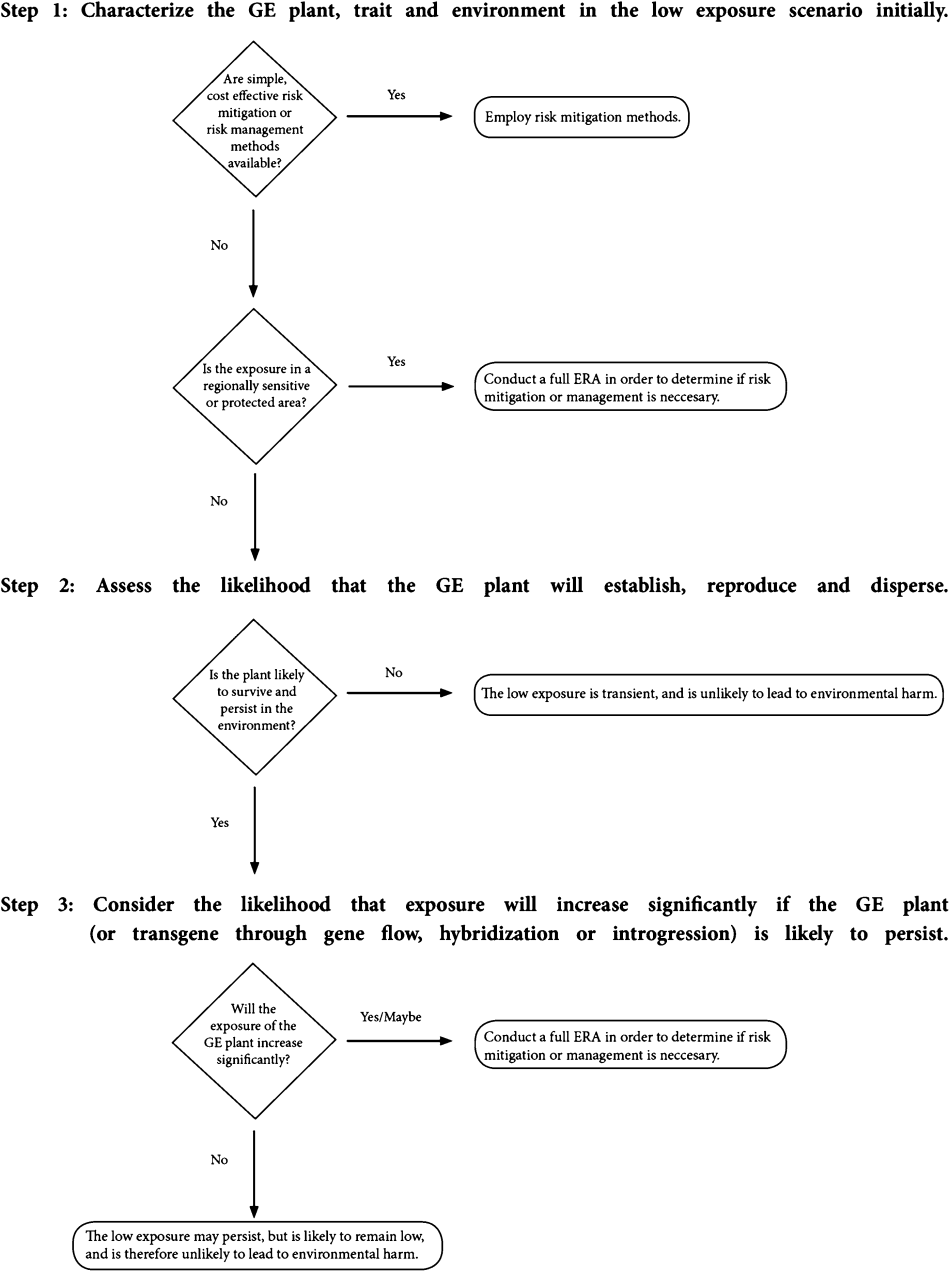
Decision tree. The decision tree illustrated here represents one example of how the step-wise process may be implemented. It is important to note that, as with all risk assessments, risk managers may decide to implement risk management strategies to mitigate an identified risk or uncertainties and/or to monitor the GE plants in order to confirm that any assumptions incorporated into the assessment are accurate

Risk assessment is undertaken in order to support decision making. In an ideal world, decision makers would be able to incorporate the results of the assessment into consideration of a range of risk mitigation options and select an option that makes wise use of resources considering the magnitude and consequences of identified risks. Although this may not always be the case (OECD 2013), the stepwise approach presented here is intended to provide a tool for risk assessors to estimate risk in the absence of exhaustive information characterizing hazards associated with a particular GE plant that has been released into the environment at low levels of exposure. A decision tree exemplifying practical application of the stepwise approach is illustrated in Fig. 1.

The occurrence of GE plants in the environment under low-exposure conditions can result from any of several scenarios. These can be deliberate plantings in the form of confined field trials, accidental but predictable introductions, such as those caused by the spillage of viable grains during transport, or relatively unpredictable introductions such as those caused by LLP in seed and grain. Depending on the specifics of the case, the amount of information available about the GE plant may be limited. In each case, risk assessors will need to respond to the needs of decision makers by providing a useful assessment in a relevant timeframe. By following the stepwise approach presented here, assessors can focus on information that is likely to be useful to decision makers. By first identifying whether the GE plant is likely to survive and persist in the environment the assessor can quickly determine how much information will be needed to assess the risk. This determination is informed primarily by an understanding of the biology of the plant and such information should be readily available in most cases. Because transient environmental exposures at low levels are likely to present negligible risks to the environment, additional data on plants that will not persist is unlikely to increase the utility of the assessment. When a GE plant, or a transgene through gene flow, is introduced into a persistent population, then the first priority for the assessment should be to determine if the level of exposure is likely to increase over time, either because the transgene frequency increases within the population or because the population is able to expand. If the exposure is likely to remain low, then a detailed assessment of potential interactions is unlikely to provide additional value in the overall estimate of risk.

### Elements of an ERA not fully addressed using the stepwise approach

Although the amount of information contained in an ERA for a GE plant varies by jurisdiction, the types of information and issues that are addressed are nearly universal. The stepwise approach focuses on addressing the ability of the plant to survive and persist in the receiving environment, which is one component of a typical ERA. Other issues that rely on extensive characterization of the particular plant and its interactions within the environment are not fully addressed because the low-exposure scenario dictates that these interactions will be rare and have very little potential to affect the overall level of risk posed by the introduction. Elements of ERA not necessarily considered under the stepwise approach include molecular characterization, NTO interactions, interactions with pests and diseases, and potential interactions with abiotic components of the environment (soil, water etc.). It is worth noting, however, that these may be considered to some degree based on available information, such as existing knowledge and familiarity with the plant, trait, environment and their interaction, during the initial characterization of the low-exposure scenario (step 1).

One other aspect of the stepwise approach that differs from a typical ERA is in relationship to comparisons with the untransformed counterpart. For most ERA involving GE plants, a comparative approach is used to identify characteristics of the GE plant that are different than the traditional counterpart, and then to characterize the risk from these differences. This is a critical component of these assessments because the ERA for the GE plant is not intended to isolate all of the risks associated with agriculture, or the use of plants in the environment; rather it is intended to identify any additional risks posed by the use of the GE plant. In the stepwise approach presented here, the initial assessment is not comparative. It requires an assessment of the ability of the GE plant to survive, persist, and increase in the environment in order to determine if the low-exposure scenario is likely to continue or whether exposure is expected to increase. Although this assessment will be heavily informed by the characteristics of the untransformed counterpart, the assessment is not comparative, per se. If it is determined using the stepwise approach that the GE plant is likely to persist, and exposure could increase, then the subsequent ERA would, of course, follow the comparative approach.

#### Limitations to the stepwise approach

Like any other risk assessment, the stepwise approach is intended to inform decision making, in this case related to GE plants under low-exposure conditions. As a result, there are times when the approach may not add value to a decision maker because of legal or regulatory requirements (OECD 2013), or, conversely, when a full ERA will be required regardless of the results of the stepwise assessment. For example, if the low-exposure scenario involves a regionally sensitive or protected area, it is likely that a full ERA will be requested in order to address the special status of the environment. It may also be the case that, under circumstances where mitigation measures are simple and cost effective, the stepwise approach (and indeed, any ERA) is unnecessary. An example of such a scenario might be a transportation accident involving the spillage of a container of GE seeds or viable grains that are not authorized for environmental release. In such cases, it is likely to be far easier to simply mitigate the spill rather than to undertake a risk assessment. Another obvious example of this would be low-exposure due to confined field trials. These are already undertaken based primarily on a series of well-defined risk management measures.

#### Advantages to the stepwise approach

The primary advantage to the stepwise approach is that it can be undertaken with only limited information on the particular GE plant involved. This means it is likely to be particularly useful to assessors when a low-exposure introduction of a GE plant occurs without an accompanying regulatory dossier. This may occur for a number of reasons, including because the GE plant is not intended for commercial introduction in the jurisdiction where the LLP occurs, or because the identity of the GE plant is not fully characterized. Under such circumstances, extensive information characterizing the plant may be difficult for assessors to acquire. In this case, the stepwise approach may be useful in determining whether the low-exposure scenario is likely to present an environmental risk so that decisions can be made regarding the pursuit of additional information, and the cost effectiveness of mitigation measures. The approach may be particularly useful under conditions where mitigation and management of risks are difficult, costly, or likely to impose a significant burden on particular stakeholders or social groups.

As with all risk assessment methods, the stepwise approach can only be useful if decision makers have sufficient discretion to incorporate the results of the assessment into their decision.
